# The Complex Relationship Between Heart Failure and Chronic Obstructive Pulmonary Disease: A Comprehensive Review

**DOI:** 10.3390/jcm14134774

**Published:** 2025-07-06

**Authors:** Luiza Elena Corneanu, Mara Sînziana Sîngeap, Victoria Mutruc, Ovidiu Rusalim Petriș, Tudor P. Toma, Victorița Șorodoc, Laurențiu Șorodoc, Cătălina Lionte

**Affiliations:** 1 Faculty of Medicine, Grigore T. Popa University of Medicine and Pharmacy, 700115 Iași, Romania; marasingeap@yahoo.ro (M.S.S.); ovidiupetris@yahoo.com (O.R.P.); iosintudor@googlemail.com (T.P.T.); victorita.sorodoc@umfiasi.ro (V.Ș.); laurentiu.sorodoc@umfiasi.ro (L.Ș.); 2 Second Internal Medicine Department, Sf. Spiridon Clinical Emergency Hospital, 700111 Iași, Romania; victoria.mutruc@yahoo.com; 3 Socola Institute of Psychiatry, 700282 Iași, Romania; 4 Department of Medicine, Lewisham and Greenwich NHS Trust, London SE13 6LH, UK

**Keywords:** heart failure, chronic obstructive pulmonary disease, overlapping syndrome, dyspnea, biomarkers, outcomes

## Abstract

The coexistence of heart failure (HF) and chronic obstructive pulmonary disease (COPD) presents a significant clinical challenge due to the common risk factors, overlapping symptoms, and complex pathophysiological interactions and mechanisms. This comprehensive review explores the bidirectional relationship between HF and COPD, emphasizing their combined impact on morbidity, mortality, and quality of life. Epidemiological data reveal that up to one-third of patients with HF also have COPD, complicating diagnosis and leading to suboptimal treatment strategies. We discuss the pathways through which each disease exacerbates the other, the limitations of the current staging systems, the diagnostic tools needed to differentiate cardiac from pulmonary symptoms, and the treatment choices. Therapeutic management requires careful integration of pharmacologic and non-pharmacologic strategies, with attention paid to potential drug interactions. Evidence from clinical trials confirms that beta-blockers can be safely used in patients with COPD and highlights the importance of multidisciplinary, patient-centered care models. Prevention strategies, including smoking cessation, vaccination, and patient education, play a critical role in improving outcomes. Finally, we identify key research gaps and calls for more inclusive clinical guidelines to address the needs of patients with this overlapping syndrome. A coordinated, evidence-based approach is essential for optimizing care and improving the quality of life of patients facing the dual burden of HF and COPD.

## 1. Introduction

Heart failure (HF) is a major and escalating global public health concern, currently affecting approximately 64 million individuals worldwide. Over the past three decades, epidemiological trends have demonstrated a marked rise in its age-adjusted prevalence, driven primarily by population aging, advances in cardiovascular therapies that improve survival following acute events, and the prolonged life expectancy of patients with chronic heart disease. However, the burden of HF is not uniformly distributed across the globe. In low- and middle-income countries, patients tend to present at younger ages and with more severe disease, a pattern likely influenced by the higher exposure to risk factors, socioeconomic disparities, and limited access to timely healthcare. These challenges often result in delayed diagnosis and suboptimal treatment, thereby exacerbating morbidity and mortality. Consequently, tailored, region-specific strategies are essential to address the evolving HF epidemic and improve outcomes in diverse healthcare settings [1,2,3,4].

Chronic obstructive pulmonary disease (COPD) remains a significant contributor to global morbidity and mortality, affecting an estimated 10–11% of adults aged 30 to 79 years. The prevalence of COPD is expected to rise substantially, with projections suggesting that the global burden could approach 600 million cases by 2050, driven by ongoing exposure to major risk factors such as tobacco smoke, air pollution, and occupational hazards. COPD’s prevalence is not uniformly distributed across regions. In high-income countries, it tends to increase with age and is generally lower among women and urban populations. In contrast, low- and middle-income countries report higher prevalence rates, particularly in the African and Eastern Mediterranean regions. As of 2019, the Western Pacific region accounted for the highest absolute number of cases, while the Eastern Mediterranean reported the lowest. These geographical and demographic variations reflect the complex interplay between environmental exposures, socioeconomic conditions, and healthcare disparities, underscoring the urgent need for context-specific prevention, diagnosis, and management strategies [5,6,7].

The coexistence of HF and COPD presents one of the most challenging situations encountered in clinical medicine. These two chronic conditions are leading causes of morbidity and mortality worldwide, especially among the aging population [8]. When they coexist, in most cases, the clinical picture becomes significantly more complex [9]. Patients with HF–COPD often experience overlapping symptoms like shortness of breath, dyspnea, fatigue, and exercise intolerance, complicating diagnosis, treatment, and management strategies [10]. The interrelationship between the heart and lungs—two organs intrinsically dependent on each other—means that dysfunction in one often exacerbates the clinical course of the other [11].

According to the Global Burden of Disease Study (2021), both HF and COPD are among the top five causes of disability-adjusted life years (DALYs) lost globally [12]. Epidemiologic studies suggest that up to 30–40% of patients with HF also have COPD, and vice versa [13]. This comorbidity is associated with worse outcomes, more frequent hospitalizations, increased healthcare costs, and reduced quality of life. Clinicians are often caught in a therapeutic balancing act—treating one disease without worsening the other [14].

Unfortunately, neither Global Initiative for Chronic Obstructive Lung Disease (GOLD) nor the European Society of Cardiology (ESC) guidelines offer a specific framework for dual disease classification. This knowledge gap underscores the need for collaborative, individualized assessment using tools like cardiopulmonary exercise testing (CPET) and comprehensive geriatric evaluation [15].

This review aims to provide a comprehensive understanding of this dual pathology. From definitions and epidemiology to diagnosis and treatment strategies, the goal is to highlight the key mechanisms and offer evidence-based guidance for optimal patient care. We will integrate findings from studies, such as the CIBIS-ELD, and international guidelines from the ESC, the American College of Cardiology/American Heart Association (ACC/AHA) and the GOLD. As we explore this topic in-depth, we aim to bridge the gap between cardiology and pulmonology, fostering a more unified and effective approach to managing these complex patients.

## 2. Definitions and Clinical Overview

### 2.1. What Is Heart Failure?

HF is a syndrome characterized by the heart’s inability to pump sufficient blood to meet the body’s metabolic demands. It typically evolves over time due to structural and/or functional cardiac disorders that lead to elevated ventricular filling pressures and/or an inadequate cardiac output at rest or during exertion [16,17]. HF is defined by the presence of characteristic signs (such as peripheral edema, hepatojugular reflux, pulmonary rales) and symptoms (including exertional or resting dyspnea and fatigue) [17].

HF is currently stratified based on the left ventricular ejection fraction (LVEF), a key parameter that guides both diagnostic and therapeutic pathways. Contemporary clinical guidelines propose a tripartite classification: HF with reduced ejection fraction (HFrEF), defined by an LVEF ≤40%; mildly reduced ejection fraction (HFmrEF), with values between 41 and 49%; and preserved ejection fraction (HFpEF), corresponding to an LVEF ≥50%. This framework acknowledges the heterogeneity of HF and supports a more tailored approach to its management [17].

HF results from a variety of causes—ischemic heart disease, hypertension, valvular disorders, cardiomyopathies, and more. It leads to neurohormonal activation (RAAS, sympathetic nervous system), fluid retention, and remodeling of the heart muscle. The ESC guidelines underscore the use of echocardiography and biomarkers for diagnosis. The cutoffs of the natriuretic peptides used for HF diagnosis are as follows: BNP (brain natriuretic peptide) > 35 pg/mL, NT-proBNP (N-terminal pro-brain natriuretic peptide) > 125 pg/mL [16]. Accurate identification of the underlying etiology—whether valvular, myocardial, pericardial, endocardial, or related to rhythm or conduction disturbances—is of paramount importance, as it determines the subsequent therapeutic approach [17].

### 2.2. What Is Chronic Obstructive Pulmonary Disease?

COPD is a heterogeneous, multifactorial pulmonary disorder characterized by chronic respiratory symptoms, such as dyspnea, chronic cough, and sputum production, resulting from abnormalities of the airways and/or alveoli. These changes lead to a persistent and progressive airflow limitation, associated with an abnormal inflammatory response of the lungs [18]. The main risk factor is long-term exposure to tobacco smoke. COPD occurs primarily in heavy smokers over the age of 40, but environmental pollutants, occupational exposures, and genetic conditions (like alpha-1 antitrypsin deficiency) also contribute. Both exacerbations and comorbidities significantly impact the disease prognosis [19].

It is estimated that 30–50% of patients with COPD remain undiagnosed until advanced stages, as symptoms are often attributed to other causes or comorbid conditions. This underdiagnosis contributes to a substantial economic burden on healthcare systems, largely due to the frequent hospitalizations related to acute exacerbations [20].

Lung function tests (spirometry) show a reduced FEV_1_/FVC ratio (<0.70 post-bronchodilator; FEV_1_—forced expiratory volume in one second; FVC—forced vital capacity). COPD staging, as highlighted in the GOLD guidelines, involves assessing the airflow limitation, the clinical symptoms, and the risk of exacerbations [18].

A key point is that the systemic inflammation in COPD can extend beyond the lungs, affecting other organs, particularly the heart [21]. This link may partly explain the high prevalence of HF in COPD patients. Conversely, left ventricular dysfunction can elevate pulmonary pressures, leading to pulmonary congestion and impaired gas exchange—exacerbating respiratory symptoms [22].

Together, HF and COPD create a vicious cycle where cardiopulmonary interactions perpetuate worsening symptoms, reduced functional capacity, and increased mortality [22]. Understanding each condition individually is essential before exploring their interplay in greater detail [9].

According to the AHA/ACC, multimorbidity is highly prevalent in patients with HF, with more than 85% of individuals having at least two additional chronic conditions. COPD is a frequently encountered comorbidity in this context, contributing to the complexity of clinical management and exerting a negative impact on prognosis. The development of specific, evidence-based recommendations for managing HF in the presence of multiple comorbidities, such as COPD, remains a significant challenge due to the current gaps in the literature [23].

In line with the GOLD 2025 report, HF is recognized as one of the most prevalent comorbidities in patients with COPD, with a reported prevalence ranging from 20% to 70% and an annual incidence of approximately 3–4% [24].

## 3. Epidemiology of HF–COPD Coexistence

### 3.1. Prevalence in the General and Aging Population

HF and COPD are increasingly prevalent in the elderly, with studies showing a significant overlap in the diagnoses among patients over the age of 65 [19]. As reported by various studies, nearly one-third of HF patients also meet diagnostic criteria for COPD [25].

The prevalence of COPD among patients with HF—when spirometry is used as the diagnostic tool for COPD—ranges between 25% and 50%. Pulmonary congestion in HF contributes to compression of the small airways, leading to airflow obstruction, which typically resolves with appropriate diuretic therapy. As a result, COPD is believed to be underdiagnosed in patients with HF [26].

On the other hand, the prevalence of HF among patients with COPD is estimated at only 5–20%. COPD acts as an independent risk factor for the development of HF—exacerbations increase the risk of acute coronary syndrome, which is a major cause of HFrEF [26].

### 3.2. Impact on Morbidity and Mortality

Although numerous risk factors have been identified and preventive strategies exist for both cardiovascular and pulmonary diseases, HF and COPD remain among the leading causes of morbidity and mortality worldwide. These conditions represent major diagnostic and therapeutic challenges, especially since they often coexist, with overlap reported in up to 30–50% of patients [21,27,28]. The coexistence of HF and COPD is associated with systemic effects, a progressive clinical course, and a poor prognosis [9,29]. It significantly impairs exercise tolerance and quality of life [10,30], and it has a multifactorial etiology, with shared risk factors such as smoking and advanced age, as well as common pathophysiological mechanisms, including chronic systemic inflammation [10,28]. The number of patients with HF–COPD overlap syndrome increases with advancing age [31]. The presence of both diseases increases the risk of hospitalization by over 50%, and the five-year survival rate declines significantly when compared to patients with only one of the conditions [26].

When HF and COPD are present together, patients tend to experience more severe symptoms and frequent exacerbations [32]. COPD can mask the early signs of HF, leading to delays in diagnosis. Similarly, cardiac medications, especially beta-blockers, are often underutilized due to fears of worsening pulmonary symptoms, despite evidence suggesting they are generally safe. A 2021 meta-analysis that included over 27,000 patients with COPD and comorbidities, including HF, demonstrated that beta-blockers, particularly cardioselective agents like bisoprolol, were associated with a reduced risk of exacerbations and all-cause mortality, which also applies to patients with HF–COPD. The therapy did not significantly impair lung function, except for a modest decline observed with propranolol. These findings support the safe and beneficial use of cardioselective beta-blockers in patients with COPD, especially those with concomitant cardiovascular disease [33]. However, the persistence of under-treatment is a significant problem, often resulting in avoidable worsening of the health status and increased readmission rates [14].

## 4. Risk Factors and Shared Etiologies

**Figure 1:**
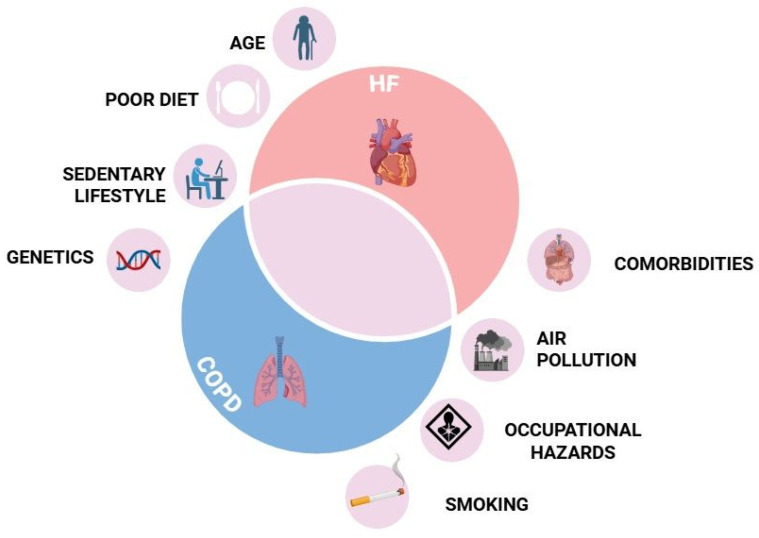
Common risk factors for HF and COPD. HF, heart failure; COPD, chronic obstructive pulmonary disease. Created with BioRender.com; accessed on 31 May 2025.

Smoking stands as the most significant shared risk factor for both diseases (Figure 1). It induces oxidative stress, systemic inflammation, and endothelial dysfunction, contributing to both airflow obstruction and myocardial remodeling. In the lungs, smoking leads to epithelial injury, ciliary dysfunction, and destruction of alveolar walls, central features of emphysema and chronic bronchitis. In the cardiovascular system, it accelerates atherosclerosis, promotes myocardial fibrosis, and increases sympathetic tone, all of which contribute to the development and progression of HF [34].

Aging, a sedentary lifestyle, poor diet (Figure 1), and chronic systemic inflammation further predispose individuals to developing both HF and COPD. With advancing age, there is a natural decline in the cardiopulmonary reserve, increased vascular stiffness, and impaired immune regulation, which together favor the onset of both pulmonary and cardiac dysfunction. Physical inactivity and unbalanced nutrition contribute to obesity, insulin resistance, and low-grade inflammation, which are associated with endothelial dysfunction, vascular remodeling, and impaired myocardial metabolism. These factors also promote respiratory muscle weakness and reduced ventilatory capacity, aggravating dyspnea and exercise intolerance [34].

Emerging data also point to shared environmental exposures such as air pollution and occupational hazards (Figure 1) as important contributors. Long-term exposure to fine particulate matter, nitrogen dioxide, and other pollutants has been associated with an increased incidence of both COPD and cardiovascular disease. These agents can penetrate deep into the alveoli and vascular endothelium, triggering oxidative stress, inflammation, and autonomic imbalance. Occupational exposures, such as dust, fumes, and chemical irritants, are similarly linked to chronic airway inflammation and elevated cardiovascular risk. The burden is disproportionately higher in low-income populations, where exposure to indoor biomass fuels and inadequate access to healthcare further increase the risk [35].

While lifestyle and environmental factors dominate the etiology, genetics also play a role in HF–COPD. Polymorphisms in genes (Figure 1) involved in inflammation and oxidative stress pathways (e.g., TNF-alpha, IL-6) are linked to both HF and COPD. These genetic variants may influence individual susceptibility by modulating the inflammatory response, tissue remodeling, or antioxidant defense mechanisms. Alpha-1 antitrypsin deficiency, though rare, is a known genetic risk factor for early-onset emphysema and can also lead to cardiac complications, particularly due to chronic hypoxemia and pulmonary vascular remodeling, which increase right heart strain [36].

Moreover, comorbidities (Figure 1) such as diabetes, chronic kidney disease, and systemic hypertension can accelerate the development and progression of both conditions [34,35]. Diabetes promotes endothelial dysfunction, myocardial fibrosis, and autonomic imbalance [37,38], while chronic kidney disease contributes to volume overload, electrolyte disturbances, and increased systemic inflammation [39,40]. Hypertension leads to increased afterload and left ventricular hypertrophy, facilitating diastolic dysfunction and HF, and it has also been associated with impaired pulmonary microcirculation and reduced lung compliance [41,42]. These overlapping risk profiles underline the importance of a holistic approach to prevention and management.

## 5. Pathophysiological Mechanisms

### 5.1. HF Exacerbates Pulmonary Pathology

The lungs and heart are interconnected in terms of more than just their proximity. HF exerts a detrimental effect on pulmonary function. The most direct influence comes from elevated left atrial and pulmonary venous pressures. When the left side of the heart fails to efficiently eject blood, it causes a backup into the lungs, resulting in pulmonary congestion and interstitial edema. This mechanical congestion impairs gas exchange, leading to symptoms like dyspnea and orthopnea, frequently misattributed solely to pulmonary disease [43].

This fluid overload can reduce lung compliance and promote bronchial hyperreactivity [44]. In HFpEF, especially, the problem is compounded by stiff ventricles that impair relaxation, further raising the pulmonary capillary wedge pressure. Even in patients without obvious signs of pulmonary disease, HF can mimic or aggravate obstructive and restrictive lung disease patterns on spirometry [36].

HF also impacts respiratory muscles. Low cardiac output reduces oxygen and nutrient delivery to the diaphragm and accessory muscles, contributing to respiratory muscle weakness and early fatigue during physical exertion [44]. Moreover, medications commonly used in HF management, like loop diuretics, can induce electrolyte imbalances (e.g., hypokalemia, hypomagnesemia), predisposing patients to arrhythmias and respiratory complications [36].

### 5.2. COPD Influences Cardiac Function

COPD, particularly in its moderate to severe forms, significantly affects cardiac function through multiple mechanisms. One of the central issues is pulmonary hypertension, which develops due to chronic hypoxia-induced vasoconstriction in the pulmonary arteries. This condition increases the afterload on the right ventricle, often leading to right-sided HF, also known as cor pulmonale. Over time, this increased pressure load can even impact left ventricular filling, compounding the dysfunction of the heart [45].

Pulmonary vascular remodeling is a hallmark of both HF and COPD, reflecting a shared pathophysiological response that contributes to the progression of pulmonary hypertension and right ventricular dysfunction. In COPD, chronic hypoxia, oxidative stress, and exposure to noxious particles activate inflammatory pathways, leading to endothelial dysfunction, perivascular infiltration of immune cells, and smooth muscle cell proliferation. Similarly, in HF, elevated left-sided filling pressures induce pulmonary venous congestion, which triggers secondary inflammation and vascular remodeling. Both conditions converge on common molecular mediators, including transforming growth factor-β (TGF-β), IL-6, and hypoxia-inducible factor-1α (HIF-1α), which promote fibroblast activation, extracellular matrix deposition, and intimal thickening. This shared inflammatory and structural remodeling process results in increased pulmonary vascular resistance and compromised gas exchange, exacerbating symptoms and perpetuating the pathophysiological interplay between the heart and the lungs. Recognizing these overlapping mechanisms is essential for identifying therapeutic targets that may benefit patients with coexisting HF–COPD [22]. 

In patients with COPD, the hemodynamic burden contributes to elevated ventricular filling pressures and atrial stretch, creating a pro-arrhythmic substrate that facilitates the development of atrial fibrillation (AF). Once established, AF further compromises cardiac output and exacerbates HF symptoms by promoting an irregular ventricular response and impairing diastolic filling. This vicious cycle may accelerate clinical deterioration, leading to increased hospitalization rates and adverse prognostic outcomes in patients with coexisting HF–COPD [46].

COPD also promotes systemic inflammation—a key driver of cardiovascular disease. Elevated levels of inflammatory markers like C-reactive protein (CRP), tumor necrosis factor-alpha (TNF-α), and interleukin-6 (IL-6) are commonly observed in COPD patients. These cytokines contribute to atherogenesis, endothelial dysfunction, and ventricular remodeling [36,45]. Another crucial pathway is through reduced oxygen delivery. Chronic hypoxemia and hypercapnia, typical in advanced COPD, place extra strain on the heart. The body compensates by increasing the cardiac output, raising the heart rate and myocardial oxygen demand—ironically worsening ischemic heart disease if present [47].

Exacerbations of COPD, often triggered by infections, also acutely worsen cardiac function. Tachycardia, hypoxia, and stress responses existing in pulmonary disease create a pro-thrombotic environment, increasing the risk of myocardial infarction and arrhythmias [35,47].

Understanding these mechanisms is vital for clinicians involved in the management of COPD patients, particularly when they show signs of cardiovascular suffering. Treating only the pulmonary aspect without considering the cardiac load can lead to suboptimal outcomes and unnecessary hospital readmissions [14].

**Figure 2:**
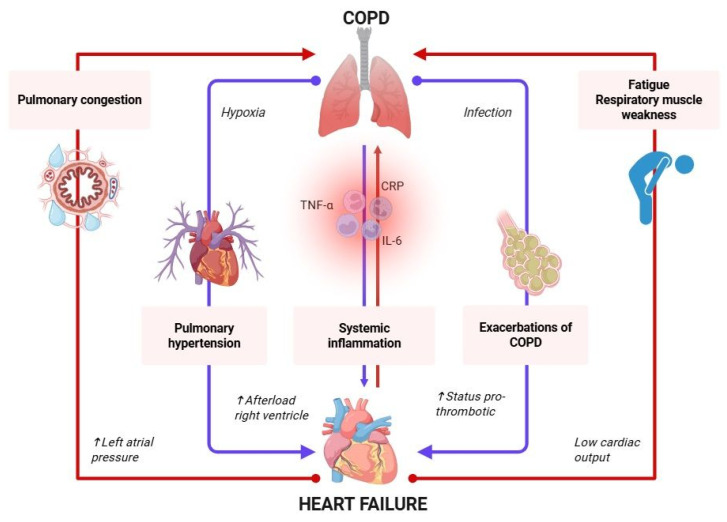
Pathophysiological mechanisms in HF–COPD. COPD, chronic obstructive pulmonary disease; CRP, C-reactive protein; TNF-α, tumor necrosis factor-α; IL-6, interleukin-6; ↑, high, elevated. Created with BioRender.com; accessed on 31 May 2025.

Thus, HF and COPD are not merely coexistent by coincidence. They share a bidirectional pathophysiological relationship (Figure 2), where deterioration in one organ amplifies dysfunction in the other. An in-depth understanding of this dynamic is essential for precise management and treatment strategies.

## 6. Disease Staging and Classification

### 6.1. NYHA and ACC/AHA/HFSA Classifications for HF

The New York Heart Association (NYHA) functional classification categorizes HF based on the degree of symptom limitation during physical activity [17,48] (Supplementary Figure S1). On the other hand, the ACC/AHA classifies HF in four stages, which emphasize the longitudinal development and escalation of the disease burden (Supplementary Figure S2) [23].

Though subjective, the NYHA staging remains an essential tool for therapeutic decisions and prognosis assessment. It is widely used in HF trials, including PARADIGM-HF [49] and EMPEROR-Reduced [50] and EMPEROR-Preserved [51].

### 6.2. GOLD Classification for COPD

GOLD provides a well-established framework for diagnosing and staging COPD based on both the spirometry and symptom burden (Supplementary Figure S2). The GOLD 2025 guidelines categorize COPD into four grades (GOLD 1–4) based on the degree of airflow limitation measured by the FEV_1_ [18].

Additionally, patients are grouped into three classes—A, B, and E (Supplementary Figure S3)—based on the symptom severity (using mMRC [52] (Supplementary Figure S1) and/or CAT scores [53]) and history of exacerbations. This multidimensional assessment helps tailor pharmacologic and non-pharmacologic therapies [18,54].

In HF–COPD patients, staging becomes more complex. Cardiac conditions can influence the spirometric values, sometimes leading to misclassification. For example, fluid overload in HF may restrict lung volumes and mask airflow limitation. That is why repeat testing after diuretic therapy or during stable periods is often recommended [15].

### 6.3. Combined Phenotypes and Implications

When HF and COPD co-occur, a combined phenotype must be considered. For instance, a GOLD 2–B COPD patient with NYHA Class III HF represents a high-risk individual requiring integrated care [15].

The interplay between these classifications directly impacts prognosis and therapy. For example, an advanced GOLD stage with preserved ejection fraction may still benefit from optimal guideline-directed medical therapy for HF if the exercise intolerance is primarily cardiac-driven. Clinicians must look beyond rigid guidelines and assess the dominant pathology at each stage of disease progression [22].

The GOLD COPD classification is based on the post-bronchodilator FEV_1_ and reflects the degree of airflow limitation. It is generally considered progressive and largely irreversible. Although treatment can improve the symptoms and reduce the frequency of exacerbations, the FEV_1_ values rarely increase sufficiently to result in a meaningful change in the GOLD functional class. For example, a patient classified as GOLD 3 is unlikely to regress to GOLD 1, although disease stabilization and improvement in quality of life can be achieved [24]. By comparison (Supplementary Figure S2), the ACC/AHA classification of HF is also considered progressive and irreversible. Although a patient in stage C may become asymptomatic with appropriate treatment, the patient nonetheless remain classified as stage C [23].

Similarly, the dynamic nature of the NYHA and ABE classifications can be observed. These staging systems may evolve over time. With effective treatment, a patient initially categorized in group E may transition to group B or even A if their symptoms and exacerbations are reduced [24], just as a patient with NYHA class III HF may improve to class II [17,23].

Recent phenotype profiling in HFpEF reveals that COPD coexists in approximately 15–20% of these patients. This comorbidity substantially increases the diagnostic complexity, as dyspnea and exercise intolerance may derive from cardiac, pulmonary, or mixed etiologies. Consequently, a comprehensive diagnostic approach, including systematic pulmonary function testing and targeted imaging, should be integrated into the routine evaluation of HFpEF. Importantly, early identification of COPD in this population allows for tailored interventions, such as bronchodilator optimization, pulmonary rehabilitation, and adjustment of diuretic therapy, which may improve both the respiratory and cardiovascular outcomes. These considerations underscore the need to intensify efforts in terms of detecting COPD among HFpEF patients to ensure accurate phenotyping and personalized, multidisciplinary management [55].

## 7. Diagnostic Challenges and Clinical Clues

### 7.1. Overlapping Symptoms

One of the greatest difficulties in managing patients with HF–COPD consists of identifying the underlying cause of shared symptoms. Dyspnea is the hallmark complaint in both diseases, but its origins differ—ventilatory inefficiency in COPD versus congestion and low cardiac output in HF. Unfortunately, the symptom presentation often overlaps to the point where distinguishing between the two becomes impossible without objective testing [18,26,56].

Chronic cough and fatigue are also shared features. Fatigue in HF stems from poor perfusion and muscle deconditioning, while in COPD, it is usually due to hypoxemia and work of breathing. Exertional symptoms are particularly intricate, as both conditions limit exercise tolerance [18,26].

Wheezing, while typically associated with COPD, can also occur in HF due to pulmonary edema. This “cardiac asthma” can lead to misdiagnosis and inappropriate use of bronchodilators [56,57]. Conversely, peripheral edema, usually a cardiac sign, can be seen in COPD patients on long-term steroids or with pulmonary hypertension [58].

### 7.2. Diagnostic Tools

A thorough diagnostic workup in HF–COPD patients is essential. Transthoracic echocardiography (TTE) is the gold standard for evaluating cardiac function [59]. It is a valuable tool for assessing the structural and/or functional abnormalities underlying HF. TTE allows for the estimation of the LVEF, the pulmonary artery systolic pressure (which is essential for identifying pulmonary hypertension, though not for determining its etiology), and the measurement of cardiac chambers dimensions, wall motion abnormalities, and valvular integrity, among other parameters [58,59].

Spirometry is the gold-standard test for the diagnosis of COPD and classifies the severity [29]; it is accessible, reproductible, non-invasive, cheap, and fast. The key indicator of airway obstruction specific to COPD is the FEV_1_/FVC ratio. According to the GOLD guidelines, a post-bronchodilator FEV_1_/FVC ratio of less than 0.7 is required for a positive diagnosis [18].

However, the medical literature recognizes that a decline in the FEV_1_/FVC ratio is a physiological consequence of aging. Thus, reliance on a fixed cutoff value may lead to overdiagnosis in the elderly and underdiagnosis in younger adults, compared to using the lower limit of normal (LLN) as a threshold. Additionally, HF is predominantly a condition of the elderly, and some patients may present airflow limitation due to aging rather than a pathological condition [28,29]. Despite their limitations, spirometric parameters such as the FEV_1_, FVC and FEV_1_/FVC ratio remain the primary tools for diagnosing and assessing the prognosis of COPD [24].

Nonetheless, it has been observed that many patients with normal spirometry still exhibit signs, symptoms, radiological changes, and even frequent exacerbations characteristic of COPD [28]. Moreover, an obstructive pattern has been documented in patients with HF, likely as a result of pulmonary congestion. Despite this, spirometry remains underutilized in patients with HF, although it is essential both for the diagnosis of COPD and for assessing the prognostic implications of the coexistence of these two conditions [60].

Lung ultrasound has emerged as a high-sensitivity, rapid, and easily accessible imaging modality that is increasingly integrated into clinical practice. It has demonstrated superior accuracy compared to conventional chest radiography in the detection of pleural effusion. In conditions characterized by reduced pulmonary aeration and increased tissue density, B-lines—vertical, hyperechoic artifacts—can be identified. These artifacts are observed in a variety of pathological states, including HF, pulmonary edema, interstitial pulmonary fibrosis, and pneumonia. The presence and quantification of B-lines provide valuable information for the diagnosis, therapeutic monitoring, and prognostic assessment of patients experiencing acute decompensation of HF [61,62,63,64].

According to the 2021 ESC guidelines, routine lung ultrasound is recommended in HF management, primarily in the context of worsening clinical status. However, there is growing interest in its incorporation into routine bedside evaluation to facilitate the early detection of subclinical pulmonary congestion, particularly when used in conjunction with transthoracic echocardiography [17].

While the application of lung ultrasound in COPD remains less well-defined, emerging evidence from recent clinical trials suggests a potential role during acute exacerbations, where B-lines may occasionally be visualized. Nonetheless, further research is warranted to establish its diagnostic and prognostic utility in this patient population [64].

Cardiac magnetic resonance imaging (CMR) represents a pivotal non-invasive modality in the differential diagnosis of dyspnea, particularly in distinguishing between cardiac and pulmonary etiologies such as HF and COPD. Its ability to accurately and reproducibly evaluate the biventricular structure and function, combined with advanced tissue characterization techniques, enables the identification of myocardial ischemia, fibrosis, or infiltrative disease that may underline HF. Moreover, CMR provides a detailed assessment of the right ventricular morphology and performance, offering critical insight in cases where pulmonary hypertension is suspected. As clinical experience and outcome data continue to expand, CMR is expected to play an increasingly central role in guiding diagnosis, risk stratification, and therapeutic decisions in patients presenting with unexplained dyspnea [65,66,67].

Complementary to CMR, speckle-tracking strain echocardiography has emerged as a valuable tool in the functional assessment of myocardial mechanics, particularly in differentiating cardiac from pulmonary causes of dyspnea. In patients with COPD, strain imaging can detect early subclinical right ventricular dysfunction, often missed by conventional echocardiographic parameters, highlighting the impact of chronic pulmonary pressure overload. Additionally, global longitudinal strain (GLS) of the left ventricle may remain impaired in HF even when the ejection fraction appears preserved, supporting the cardiac etiology of the symptoms. Thus, strain echocardiography enhances diagnostic precision in complex cases where clinical and standard imaging findings are inconclusive, reinforcing its role in the comprehensive evaluation of dyspnea in patients with suspected HF–COPD overlap [68].

**Figure 3:**
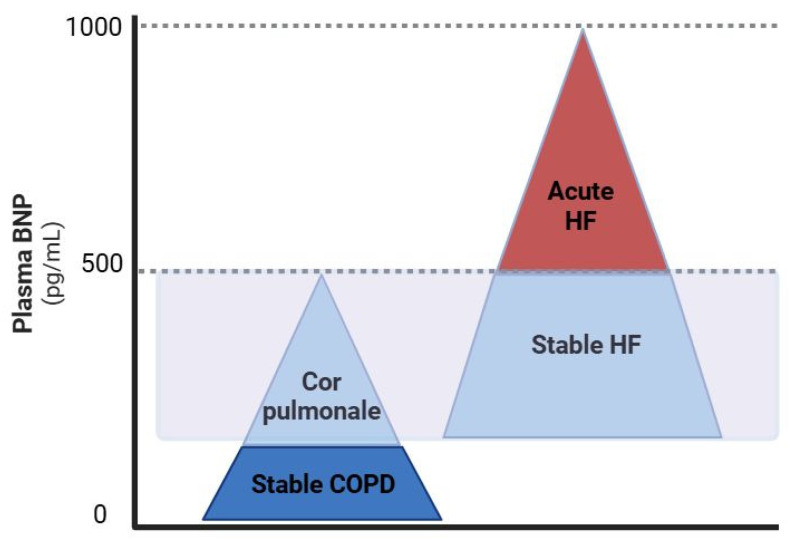
Overlap in the BNP levels in patients with HF and COPD. COPD, chronic obstructive pulmonary disease; HF, heart failure. Created with BioRender.com; accessed on 11 June 2025.

Biomarkers like natriuretic peptides (BNP and NT-proBNP) are valuable in differentiating HF from other causes of dyspnea. Their levels serve not only as a diagnostic tool but also assist in determining the urgency of care, with specific thresholds guiding decisions on hospitalization and further cardiac evaluation [69]. Elevated levels (Table 1) (BNP ≥ 35 pg/mL, NT-proBNP ≥ 125 pg/mL—for ambulatory patients; BNP ≥ 100 pg/mL, NT-proBNP ≥ 300 pg/mL—in hospitalized patients with acute decompensation) strongly suggest a cardiac etiology [16], although these markers can also be mildly raised in patients with COPD, with increased levels observed in approximately 50% of cases, particularly during exacerbations or in the presence of cor pulmonale (Figure 3) [26,70,71]. Patients with natriuretic peptide levels falling within the so-called “gray zone” represent a diagnostic challenge, as the values are neither sufficiently low to exclude heart failure nor high enough to confirm it with certainty. In such cases, a comprehensive clinical evaluation is recommended, including echocardiographic assessment and consideration of alternative contributors to natriuretic peptide elevation, such as age, renal dysfunction, or atrial fibrillation. These patients require careful monitoring, and decisions regarding hospitalization or close outpatient follow-up should be guided by the overall clinical context and risk profile [69].

Although clinical studies have varied in the design and objectives regarding the use of natriuretic peptides in different categories of HF patients, the use of BNP/NT-proBNP for guiding pharmacological treatment in HF has been associated with reduced mortality and fewer hospitalizations, particularly in patients under 75 years of age with HFrEF [72].

Despite advances in proteomic research identifying several potential biomarkers, including CC16 protein, none have yet been implemented in clinical practice. Currently, there are no reliable biomarkers capable of predicting COPD onset prior to functional decline, and the disease severity continues to be defined primarily by the FEV_1_ [73,74,75]. According to the GOLD 2025 report, the peripheral blood eosinophil levels currently serve as a useful biomarker to help identify individuals with COPD who are at increased risk of exacerbations and who are more likely to derive clinical benefit from preventive therapy with inhaled corticosteroids [24].

Other useful tests include chest X-ray (to detect pulmonary congestion), arterial blood gases (ABGs), and CPET, which can help determine whether exercise limitation is cardiac or pulmonary in origin [15,20,59].

Table S1 (Supplementary Materials) summarizes the key diagnostic features and differentiating criteria between HF and COPD, based on the clinical presentation, biomarkers, imaging, and functional assessment.

## 8. Therapeutic Management Strategies

### 8.1. Non-Pharmacologic Interventions

Beyond medications, non-pharmacologic strategies play an important role. Cardiac rehab provides benefits for HF patients. Similarly, pulmonary rehabilitation has been shown to improve exercise capacity, reduce hospitalizations, and enhance quality of life in COPD patients. For those with both conditions, combined rehabilitation programs targeting endurance, strength and breathing techniques can lead to substantial functional improvement [45,57].

Cardiac rehabilitation (CR) is increasingly recognized as a cornerstone of the comprehensive management of HF, with strong guideline support for its routine implementation. Exercise-based CR programs have demonstrated substantial benefits, including improved quality of life, enhanced functional capacity, and a reduction in hospital readmissions, even though a consistent mortality-related benefit has yet to be established. These programs adopt a multidisciplinary, patient-centered approach that combines supervised physical activity with education, lifestyle modification, and psychosocial support. Despite these advantages and the Class I recommendations in international guidelines, CR remains significantly underutilized, with participation rates often below 20% due to barriers such as limited referral, patient awareness, and logistical constraints. To improve accessibility and adherence, alternative delivery models such as home-based, digital, or hybrid programs are encouraged. Importantly, CR is safe across a broad spectrum of HF patients, including those with preserved ejection fraction, and should be integrated within a wider continuum of care, alongside pharmacologic treatment and long-term disease management strategies. As such, CR plays a vital role in both secondary prevention and long-term stabilization in patients with heart failure [76].

Pulmonary rehabilitation (PR) is recognized as a key component of the comprehensive management of patients with severe and very severe COPD. According to the findings of the systematic review and meta-analysis by He et al. (2023) [77], exercise-based PR significantly improves exercise tolerance, reduces dyspnea, and enhances health-related quality of life in this patient population. These benefits support the inclusion of PR as a standard non-pharmacological intervention, even in advanced stages of COPD. Despite some variability in the study quality, the evidence strongly favors its implementation in routine clinical practice [77].

Effective implementation of cardiopulmonary rehabilitation (CPR) in patients with HF–COPD overlap requires close collaboration between cardiologists and pulmonologists, ideally within multidisciplinary clinics that facilitate integrated, disease-specific care [78].

Exercise-based CPR has emerged as a key therapeutic strategy in patients with coexisting HF and COPD. These individuals often exhibit severe limitations in their exercise capacity due to combined impairments in ventilatory function, gas exchange, peripheral muscle performance, and hemodynamic response. Structured rehabilitation programs incorporating aerobic training (e.g., walking, cycling), resistance exercises, and inspiratory muscle training have demonstrated safety, tolerability, and significant clinical benefits in this population. Such interventions contribute to improved functional status, reduced hospital readmissions, and enhanced quality of life. Additionally, adjunctive use of non-invasive ventilation during exercise sessions may further optimize physiological responses and exercise tolerance. Current evidence highlights the importance of delivering these interventions within integrated care models involving both cardiologists and pulmonologists, thereby ensuring a comprehensive and individualized therapeutic approach for patients facing the dual burden of HF and COPD [79].

Long-term oxygen therapy (LTOT) is indicated in COPD patients with chronic hypoxemia and may benefit HF patients with overlapping hypoxic symptoms. However, oxygen must be prescribed carefully, as hyperoxia can suppress the hypoxic drive in COPD and worsen carbon dioxide (CO_2_) retention [18,57].

Vaccination against influenza, pneumococcus and COVID-19 is strongly recommended for both HF and COPD patients due to their increased risk of severe infections [17,18].

Patient education on recognizing the early symptoms of exacerbation, adherence and lifestyle changes—including smoking cessation and nutrition—remains the cornerstone of chronic disease management [16].

### 8.2. Pharmacologic Management

Managing both HF and COPD simultaneously requires a careful balancing act. Many drugs that are mainstays for one condition may either be contraindicated or used cautiously in the other. However, modern evidence suggests that, with proper selection and monitoring, most of these therapies can coexist safely and even synergistically [80].

**Figure 4:**
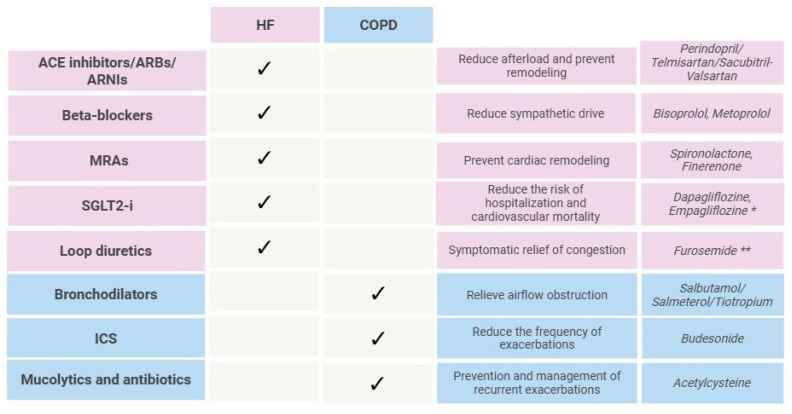
Guideline-directed treatment in HF and COPD. HF, heart failure; COPD, chronic obstructive pulmonary disease; ACE, angiotensin-converting enzyme; ARBs, angiotensin II receptor blockers; ARNIs, angiotensin receptor-neprilysin inhibitors; MRAs, mineralocorticoid receptor antagonists; SGLT2-i, sodium–glucose co-transporter 2; ICS, inhaled corticosteroids. Created with BioRender.com; accessed on 9 June 2025. * Recent studies suggest that SGLT2-i may have protective effects in COPD [81,82,83]. ** Administration of nebulized furosemide may contribute to the improvement and normalization of vital signs and respiratory function in individuals with COPD [84].

The figure (Figure 4) below outlines the recommended therapeutic strategies for HF and COPD according to current clinical guidelines [16,17,18,45].

The available data indicate that medical treatments aligned with clinical guidelines substantially reduce both mortality and hospitalization rates in patients with HF, especially those with reduced ejection fraction, and are consequently considered highly cost-effective therapeutic strategies [1].

Concerns often arise around the use of beta-blockers in COPD due to the risk of bronchospasm. However, it has been shown that cardioselective beta-blockers do not significantly worsen lung function and are under-prescribed in this group [85]. The use of beta-blocker therapy, a cornerstone of the management of HF, is considered safe and appropriate even in the presence of concomitant COPD, provided that cardioselective agents are used. Moreover, oxygen therapy represents a fundamental component of the acute management of both decompensated HF and COPD exacerbations, contributing to improved oxygenation and symptom relief [24].

The available evidence consistently supports the preferential use of β_1_-selective beta-blockers, such as bisoprolol, metoprolol, and nebivolol, in patients with HF and coexisting COPD. These agents have demonstrated a favorable safety profile, maintaining their well-established benefits in reducing morbidity and mortality in HF, without significantly affecting pulmonary function or increasing the risk of COPD exacerbations. Conversely, non-selective beta-blockers, by antagonizing β_2_-receptors in the bronchial smooth muscle, are more likely to induce bronchospasm or reduce forced expiratory volumes, particularly during initiation or up-titration [86,87]. As a result, clinical guidelines recommend initiating treatment with cardioselective agents, with gradual dose escalation under close monitoring to optimize the cardiovascular outcomes while minimizing the potential respiratory compromise [18]. This individualized approach is essential in managing patients with dual pathology, ensuring both efficacy and safety. Similarly, inhaled beta-agonists may have minor cardiac effects (tachycardia), but in well-controlled HF, they are usually tolerated. ICS used in COPD is linked to a higher pneumonia risk, which risk may be amplified in HF patients with already compromised pulmonary defenses. The key lies in selecting patients based on the eosinophil count, exacerbation history, and clinical phenotype [47].

Recent findings suggest that ICS, widely used in the treatment of COPD, may also confer cardiovascular benefits. Data indicate a potential association between ICS use and a reduced incidence of coronary events, particularly among patients with early or less severe forms of COPD. These favorable outcomes may be attributed to the systemic anti-inflammatory effects of ICS, targeting pathophysiological mechanisms common to both pulmonary and cardiovascular diseases. Nonetheless, clinical decisions should be individualized, as long-term safety and the balance between therapeutic efficacy and risks, such as increased susceptibility to respiratory infections, remain important considerations [88,89].

Long-acting bronchodilators, including long-acting β_2_-agonists (LABAs) and long-acting muscarinic antagonists (LAMAs), are cornerstone therapies in the management of chronic obstructive pulmonary disease. However, their cardiovascular safety profile has been subject to ongoing scrutiny. Current evidence suggests a transient increase in the cardiovascular risk, particularly within the first month of treatment initiation, which may reflect hemodynamic shifts or autonomic modulation associated with bronchodilation. Notably, this elevated risk appears to attenuate with continued use, implying a potential adaptation over time. Moreover, therapeutic regimens that incorporate inhaled corticosteroids alongside bronchodilators may confer a more favorable cardiovascular profile compared to bronchodilator monotherapy. These observations underscore the importance of individualized risk assessment and close cardiovascular monitoring during the early phases of treatment, especially in patients with pre-existing cardiac comorbidities [90,91].

Polypharmacy, drug–drug interactions and adherence are major issues. Close monitoring of renal function, electrolytes and vital signs is essential. Medication reconciliation at every healthcare encounter is also crucial to avoid therapeutic duplications and omissions [21].

### 8.3. New Perspectives

Finerenone, a novel non-steroidal selective MRA, has emerged as a promising therapeutic agent in the management of HFmrEF or HFpEF, including in patients with coexisting COPD. Through its potent anti-inflammatory and antifibrotic mechanisms, finerenone may attenuate systemic inflammation and pulmonary vascular remodeling, key contributors to the pathophysiology of both conditions. Preliminary evidence indicates that it may confer clinical benefit while maintaining a favorable safety profile, with minimal impact on the serum potassium levels and no significant increase in respiratory adverse events. These findings underscore the need for further research to clarify its role in the therapeutic strategy for patients with concurrent HF and COPD [92].

Recent evidence suggests that SGLT2-i may confer protective effects in patients with coexisting type 2 diabetes and COPD, extending their therapeutic impact beyond glycemic control. Data from large real-world analyses demonstrate a lower incidence of moderate to severe COPD exacerbations, fewer hospital admissions, and reduced all-cause mortality in patients treated with SGLT2-i. These benefits may reflect the pleiotropic mechanisms of the drug, including anti-inflammatory properties, mild diuretic effects, and potential improvement in pulmonary congestion, positioning SGLT2-i as a promising adjunct in the multidisciplinary management of patients at the intersection of metabolic and respiratory disease [81,82,83].

Building upon these findings, further clinical and mechanistic studies have highlighted the broader role of SGLT2 inhibitors in patients with overlapping HF–COPD. In randomized trials such as DAPA-HF, the cardioprotective effects of dapagliflozin were maintained irrespective of the COPD status, with consistent reductions in HF hospitalizations and cardiovascular mortality. These outcomes are believed to stem from a constellation of pleiotropic actions, ranging from attenuation of oxidative stress and systemic inflammation to improved endothelial function and modulation of pulmonary vascular stiffness [93]. Moreover, reductions in the incidence of respiratory events such as pulmonary edema and sleep apnea have been observed in patients receiving SGLT2-i, reinforcing their therapeutic potential in multimorbid populations [94]. Taken together, these observations support the integration of SGLT2 inhibitors as a valuable component of the holistic management of patients with concomitant cardiovascular and pulmonary disease [95].

Also, glucagon-like peptide-1 receptor agonists (GLP-1 RAs), beyond their established metabolic effects in type 2 diabetes, may exert protective actions in patients with coexisting COPD. Observational data indicate a statistically significant association between GLP-1 RA use and a reduced incidence of both moderate and severe COPD exacerbations when compared to other glucose-lowering agents. The proposed mechanisms include anti-inflammatory effects on the airway epithelium, modulation of oxidative stress, and improved pulmonary endothelial function, all of which may contribute to enhanced respiratory outcomes. While randomized controlled trials are still warranted to confirm causality, these findings highlight the potential of GLP-1 RAs to address both the metabolic dysregulation and the pulmonary disease burden in this complex patient population [96,97].

## 9. Evidence from Clinical Trials and Meta-Analyses

The dual burden of HF and COPD has been a focus of numerous observational studies, randomized controlled trials, and meta-analyses (Table 2) over the past two decades. While most landmark trials have historically excluded patients with significant comorbidities, a growing number of studies now acknowledge the importance of this overlap population [26].

In the PARAGON-HF trial, approximately one in seven patients with HFpEF had coexisting COPD, and these individuals exhibited significantly worse functional status, higher NYHA classes, and poorer quality-of-life scores compared to those without COPD. They also demonstrated greater right ventricular enlargement, elevated inflammatory markers, and increased serum creatinine. Importantly, after adjustment for confounders, COPD remained independently associated with higher rates of HF hospitalization, cardiovascular mortality, and all-cause death, attributable to a ~50% increased risk of the composite primary outcome and mortality [98].

In the PARADIGM-HF study, the coexistence of COPD in patients with HFrEF was associated with a distinct clinical profile and less favorable outcomes. The presence of COPD correlated with a reduced use of beta-blockers, a lower likelihood of achieving target doses, and an overall attenuation in the benefits of standard HF therapies. Despite these challenges, sacubitril/valsartan remained effective across the subgroups, including those with COPD, suggesting that optimized neurohormonal blockade retains prognostic value even in the context of comorbid pulmonary disease [99].

The EVEREST trial found that in patients hospitalized for worsening HFrEF, the presence of COPD was associated with a higher symptom burden, more frequent respiratory comorbidities, and increased in-hospital and post-discharge mortality. The study highlighted that COPD contributes to a more complex clinical presentation and therapeutic management, often leading to underutilization of guideline-directed medical therapy due to concerns about respiratory side effects [100].

The CHARM (Candesartan in Heart failure: Assessment of Reduction in Mortality and morbidity) trial, although focused on HF, provided subgroup analyses indicating that the benefits of angiotensin receptor blockers (ARBs) extend to patients with COPD, without increasing pulmonary complications [101].

Similarly, the CIBIS-ELD trial studied the tolerability of beta-blockers in elderly HF patients, including those with COPD. The results were clear—cardioselective beta-blockers such as bisoprolol and nebivolol are well tolerated and significantly reduce mortality and hospitalizations, even in those with mild-to-moderate COPD [85].

On the respiratory side, the TORCH (Towards a Revolution in COPD Health) study demonstrated that inhaled corticosteroids combined with long-acting beta-agonists reduce COPD exacerbations and modestly impact mortality. Subgroup analyses suggested that these benefits are not diminished in patients with coexisting cardiovascular disease [102].

These findings highlight that therapeutic inertia or under-treatment due to comorbidity fears is both common and dangerous. Patients benefit most when clinicians apply evidence-based interventions in a personalized, monitored way, rather than withholding therapies out of caution.

## 10. Potential Drug Interactions and Contraindications

The coexistence of HF and COPD often leads to a “pharmacological standoff.” Clinicians worry that treating one condition may worsen the other. Nowhere is this more evident than with beta-blockers and bronchodilators [31].

Beta-blockers, particularly non-selective ones (like propranolol), are traditionally avoided in COPD due to the risk of bronchoconstriction. However, cardioselective agents (like bisoprolol and nebivolol) predominantly act on the beta-1 receptors in the heart and have minimal impact on the beta-2 receptors in the lungs. Randomized controlled trials (e.g., SENIORS study) confirm that these agents are not only safe in COPD but also reduce mortality in HF [85,103].

Bronchodilators, especially LABAs and LAMAs, improve airflow and quality of life in COPD. Yet, they carry some cardiovascular risks, including tachyarrhythmias and increased myocardial oxygen demand, which are particularly concerning in decompensated HF. Close monitoring and starting with the lowest effective dose can mitigate these risks [104].

Systemic corticosteroids, while often necessary during COPD exacerbations, can exacerbate fluid retention, hypertension and glucose intolerance, worsening HF. Short courses at the lowest effective dose, ideally under pulmonologist supervision, are preferred. Long-term use should be avoided unless absolutely necessary [105,106].

Diuretics, the cornerstone of HF symptom management, can lead to electrolyte imbalances (e.g., hypokalemia, hyponatremia) that increase the risk of arrhythmias—already a concern in hypoxic COPD patients. Potassium-sparing agents and careful lab monitoring can help balance this risk [107].

Polypharmacy is often unavoidable, but using medication reconciliation tools, pharmacist collaboration and electronic alert systems can help reduce adverse events. Risk–benefit analysis tailored to each patient’s phenotype, stage, and comorbidities is critical to successful dual disease management [1].

## 11. Impact on Prognosis and Quality of Life

The presence of both HF and COPD worsens nearly every aspect of a patient’s health trajectory. Each disease alone is associated with reduced exercise tolerance and quality of life, but together, they compound the burden [28].

Patients with HF-COPD face the following: (i) higher risk of hospitalization—patients with coexisting two diseases are two to three times more likely to be readmitted within 30 days post-discharge compared to single-diagnosis individuals; (ii) increased mortality—the combination of diseases raises all-cause mortality by over 50% compared to either condition alone; and (iii) worse response to therapy—the treatment response, particularly to beta-blockers or bronchodilators, may be blunted in dual-diagnosis patients due to therapeutic hesitancy or adverse effects [28].

This burden also extends to caregivers and health systems, with frequent emergency visits, prolonged hospital stays, and increased need for post-acute care or rehabilitation [108].

Beyond physical health, the psychological impact is profound. Anxiety and depression are more common in patients with HF–COPD due to the constant breathlessness, medication burden and fear of exacerbations. Studies suggest that nearly 40–50% of these patients meet criteria for at least mild depressive symptoms [109].

Cognitive decline, driven by chronic hypoxia and reduced cerebral perfusion, adds another layer of complexity, affecting medication adherence and self-management capacity [109].

Addressing these issues requires more than medications—it demands compassionate, patient-centered care. This includes regular psychological screening, access to mental health services and supportive interventions such as motivational interviewing, peer support groups and telehealth follow-ups [108].

The focus should be on maximizing function, not just extending life. Encouraging goal-setting, shared decision-making, and quality-of-life assessments helps personalize care and empower patients [108].

The Kansas City Cardiomyopathy Questionnaire (KCCQ) and the St George’s Respiratory Questionnaire (SGRQ) are widely validated patient-reported outcome measures used to assess the symptom burden, functional status, and health-related quality of life in HF and COPD, respectively [110,111]. Both tools are endorsed by international guidelines (KCCQ by the ESC for HF and SGRQ by the GOLD for COPD) as reliable instruments for clinical assessment. In addition to their prognostic utility, they are sensitive to changes over time and are increasingly employed to evaluate the treatment response, supporting personalized care and long-term disease management [17,18].

## 12. Role of Multidisciplinary Care

Given the intertwined pathophysiology and overlapping treatment needs, the ideal care model for HF–COPD overlap involves multidisciplinary collaboration. Multispecialty clinics—especially those that integrate cardiologists, pulmonologists, nurse specialists and physiotherapists—are proving to be the gold standard for managing these complex patients [108].

The United Kingdom’s BREATHE program demonstrates that such coordinated care significantly reduces hospital readmissions, improves medication adherence, enhances patient satisfaction, and increases functional capacity through synchronized rehabilitation plans [112].

This type of clinic offers comprehensive care, from diagnostics to medication titration, nutrition counseling, and end-of-life planning, all in one coordinated environment. Electronic health records allow seamless communication between providers, reducing duplication and medical errors [112].

Case managers and nurse navigators also play an essential role, offering consistent follow-up and helping patients manage appointments, medications, and lifestyle changes [112].

This team-based approach is particularly critical for elderly patients with multiple comorbidities, cognitive impairment, or socioeconomic barriers. By shifting from fragmented, specialist-centered care to collaborative, person-centered care, we can dramatically improve both outcomes and quality of life [112].

## 13. Prevention and Patient Education

**Figure 5:**
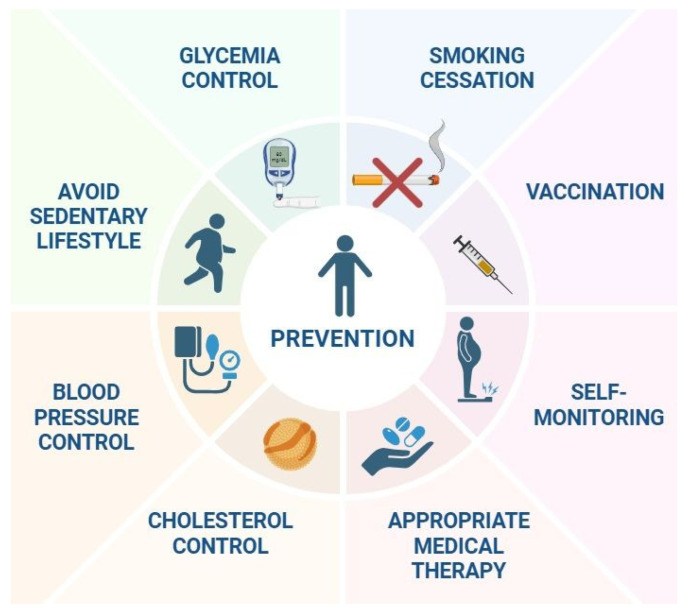
Prevention of HF–COPD. Created with BioRender.com; accessed on 8 June 2025.

When it comes to HF and COPD, prevention and education are not optional—they are lifesaving. Smoking cessation (Figure 5) remains the single most impactful intervention for preventing the progression of both diseases. Counseling, nicotine replacement therapy, and pharmacologic aids like varenicline or bupropion must be offered routinely [16].

Vaccination (Figure 5) against influenza, pneumococcus, respiratory syncytial virus (RSV), and COVID-19 helps prevent exacerbations that can trigger decompensation in either condition. Many hospitalizations and deaths can be avoided with simple immunization strategies, as supported by both the ESC and GOLD guidelines [17,18].

Self-monitoring (Figure 5) is also essential. Patients should be trained to recognize signs of fluid overload (e.g., weight gain, edema); monitor daily symptoms like dyspnea or sputum color; use peak-flow meters or home spirometry if available; and understand their medication regimen and side effects [113].

Mobile health apps and remote monitoring technologies are increasingly valuable. Some systems allow patients to upload daily vitals, medication logs, and symptom scores, which healthcare teams can review in real time [113].

Education must be ongoing and tailored to each patient’s literacy level, language, and cultural context. Group education sessions, multimedia tools, and family involvement improve retention and engagement [113].

Empowered patients are better equipped to manage their illness, prevent exacerbations, and seek timely care—ultimately improving outcomes and reducing costs [113].

Preventing HF requires a comprehensive approach focused on the early identification and management of shared risk factors (Figure 5) with other chronic diseases, particularly COPD. As highlighted in the position paper by the Heart Failure Association in collaboration with the European Association of Preventive Cardiology, common risk factors such as hypertension, diabetes mellitus, dyslipidemia, smoking, sedentary habits and obesity play a pivotal role in the development of both conditions. Targeted interventions aimed at controlling these modifiable factors through lifestyle changes and appropriate medical therapy not only reduce the incidence of HF but also improve overall cardiopulmonary health. Moreover, the recognition and integrated management of comorbidities like COPD are essential to optimizing preventive strategies and improving long-term outcomes in at-risk populations [114].

## 14. Future Directions and Research Gaps

Despite advances, significant knowledge gaps remain. There are no definitive guidelines specifically addressing the HF–COPD overlap and most clinical trials still exclude this subgroup. Future research must focus on the following:

- Developing integrated risk models that combine cardiac and pulmonary parameters;
- Identifying biomarkers to distinguish the cardiac versus respiratory origin of symptoms;
- Testing dual-acting therapies that benefit both conditions without adverse cross-effects;
- Understanding the genetic and molecular basis of overlap phenotypes [60].

Furthermore, artificial intelligence and machine learning may help predict exacerbations, guide therapy adjustments, and personalize care plans [113].

On the therapeutic front, SGLT2 inhibitors and GLP-1 receptor agonists are emerging as promising options with anti-inflammatory and cardiorespiratory benefits, though more data are needed [115]. Also, finerenone represents a promising future direction for the integrated treatment of patients with coexisting HF–COPD due to its dual anti-inflammatory and antifibrotic effects [92].

Investment in translational research, multicenter trials, and real-world studies will be critical to fill these gaps. Most importantly, a paradigm shift is needed, from isolated disease management to an integrated syndrome-oriented approach to integrated care [114].

## 15. Conclusions

The co-occurrence of HF and COPD is far more than a chance event—it is a clinical challenge that demands thoughtful, coordinated, and evidence-based care. Together, these conditions amplify each other’s symptoms, complicate diagnosis, and limit treatment options. Yet, with proper understanding, multidisciplinary teamwork, and a commitment to patient-centered care, the outcomes can be improved significantly.

From shared risk factors and pathophysiology to diagnostic dilemmas and therapeutic intersections, this review has explored the many facets of the HF–COPD overlap. Clinicians must stay informed, proactive, and flexible, utilizing current evidence, clinical guidelines, and collaborative models to deliver the best care possible.

Cardioselective beta-blockers, particularly bisoprolol and nebivolol, are regarded as safe and effective in patients with coexisting HF–COPD. Inhaled bronchodilators and corticosteroids can be used judiciously, especially in patients with frequent exacerbations or eosinophilic airway inflammation. Emerging therapies such as SGLT2 inhibitors and finerenone offer additional benefits through their anti-inflammatory and organ-protective properties. A personalized, phenotype-guided therapeutic strategy is essential to optimize outcomes while minimizing adverse effects in this complex patient population.

Importantly, there is a growing need for the development of harmonized clinical guidelines that bridge the gap between cardiology and pulmonology, offering coherent strategies for risk stratification, diagnosis, and treatment. Such unified guidance would enhance consistency in practice, promote interdisciplinary synergy, and ultimately benefit a population often underserved by disease-specific protocols.

Ultimately, the goal is not just to treat diseases but to treat people. And for the millions living with both HF and COPD, this approach can make all the difference.

## Figures and Tables

**Table 1:** Recommended natriuretic peptide cutoffs for acute HF diagnosis [70].

	BNP			NT-proBNP			
	HF Unlikely	Gray Zone	HF Likely	HF Unlikely	Gray Zone	HF Likely	
**Non-acute setting**, patient with mild symptoms	**<35**	**35–150**	**>150**	**<125**	**125–600**	**>600**	
**Acute setting**, patient with acute dyspnea	**<100**	**100–400**	**>400**	**<300**			Age **<50**
				**300–450**	**300–900**	**300–1800**	Age **50–75**
				**>450**	**>900**	**>1800**	Age **>75**

**Table 2:** Summary of clinical trials involving HF, COPD, and HF–COPD.

Trial	Objective	No. Patients	Main Findings	HF–COPD Data Availability
**PARAGON-HF**	Efficacy of sacubitril/valsartan versus valsartan in HFpEF patients (LVEF ≥ 45%)	4822	No significant reduction in total HF hospitalizations and CV death vs. comparator; potential benefit in specific subgroups (women, LVEF < 57%)	No direct data; patients with severe lung disease were excluded
**PARADIGM-HF**	Sacubitril/valsartan versus enalapril in patients with HFrEF (LVEF ≤ 40%)	8442	Significant reduction in mortality and HF hospitalizations with sacubitril/valsartan; well tolerated, including in patients with COPD	Partial data available; subgroup analyses showed similar efficacy and tolerability in COPD patients
**EVEREST**	Impact of tolvaptan in hospitalized patients with HF (LVEF ≤ 40%)	4133	Early symptomatic improvement; did not improve long-term mortality or morbidity	No direct data; COPD subgroup analyses revealed greater all-cause mortality
**CHARM**	Effects of candesartan in patients with HF	7601	Reduced CV death and HF hospitalizations; benefits were consistent across subgroups (CHARM overall/low LVEF/preserved)	COPD patients were included; candesartan was effective and safe
**CIBIS-ELD**	Tolerability of bisoprolol versus carvedilol in elderly (age ≥ 65) HF (LVEF ≤ 45%) patients with and without COPD	883	Both beta-blockers were tolerated; bisoprolol showed better tolerability in patients with moderate COPD	Directly assessed tolerability in patients with HF-COPD; bisoprolol better tolerated
**TORCH**	Impact of salmeterol/fluticasone on mortality and exacerbation rate in patients with COPD	6112	Reduced exacerbations; did not significantly affect overall mortality	Primarily a COPD trial; included patients with HF

## Data Availability

Not applicable.

## Supplementary Materials

The following supporting information can be downloaded at https://www.mdpi.com/article/10.3390/jcm14134774/s1, Figure S1: NYHA versus mMRC classification. NYHA, New York Heart Association; HF, heart failure; mMRC, modified Medical Research Council, dyspnea scale. (Created with Biorender.com/; accessed on 31 May 2025). Figure S2: ACC/AHA/HFSA Heart Failure staging, GOLD classification. Comparison. COPD, Chronic Obstructive Pulmonary Disease; FEV1, forced expiratory volume in one second. (Created with Biorender.com/; accessed on 9 June 2025). Figure S3: GOLD ABE classification. mMRC, modified Medical Research Council, dyspnea scale; CAT, COPD assessment test. (Created with Biorender.com/; accessed on 31 May 2025). Table S1: Key Diagnostic Tools and Differential Features: HF vs. COPD.

## Abbreviations

The following abbreviations are used in this manuscript:

HF	Heart failure
COPD	Chronic obstructive pulmonary disease
DALYs	Disability-adjusted life years
GOLD	Global Initiative for Chronic Obstructive Lung Disease
ESC	European Society of Cardiology
CPET	Cardiopulmonary exercise testing
ACC/AHA	American College of Cardiology/American Heart Association
LVEF	Left ventricular ejection fraction
HFrEF	Heart failure with reduced ejection fraction
HFmrEF	Heart failure with mild reduced ejection fraction
HFpEF	Heart failure with preserved ejection fraction
RAAS	Renin–angiotensin–aldosterone system
BNP	Brain natriuretic peptide
NT-proBNP	N-terminal–pro-brain natriuretic peptide
FEV1	Forced expiratory volume in one second
FVC	Forced vital capacity
TNF-α	Tumor necrosis factor-α
IL-6	Interleukin-6
AF	Atrial fibrillation
CRP	C-reactive protein
TGF-β	Transforming growth factor β
HIF-1α	Hypoxia inducible factor 1α
NYHA	New York Heart Association
mMRC	Modified Medical Research Council
CAT	COPD assessment test
TTE	Transthoracic echocardiography
LLN	Lower limit of normal
CMR	Cardiac magnetic resonance
GLS	Global longitudinal strain
ABGs	Arterial blood gases
CR	Cardiac rehabilitation
PR	Pulmonary rehabilitation
CPR	Cardiopulmonary rehabilitation
LTOT	Long-term oxygen therapy
CO2	Carbon dioxide
ACE	Angiotensin-converting enzyme
ARBs	Angiotensin II receptor blockers
ARNIs	Angiotensin receptor–neprilysin inhibitor
MRAs	Mineralocorticoid receptor antagonists
SGLT2-i	Sodium–glucose co-transporter 2 inhibitor
ICS	Inhaled corticosteroids
LABA	Long-acting beta-agonists
LAMA	Long-acting antimuscarinics
GLP-1 RA	Glucagon-like peptide-1 receptor agonist
KCCQ	Kansas City Cardiomyopathy Questionnaire
SGRQ	St George’s Respiratory Questionnaire
RSV	Respiratory syncytial virus
