# Supplementary material for: The Complex Relationship Between Heart Failure and Chronic Obstructive Pulmonary Disease: A Comprehensive Review
Source: J Clin Med. 2025 Jul 6;14(13):4774. doi: 10.3390/jcm14134774 (PMC12250576; doi:10.3390/jcm14134774)
Supplement: Supplementary file 1 [file jcm-14-04774-s001.zip › jcm-3731840-supplementary.pdf]

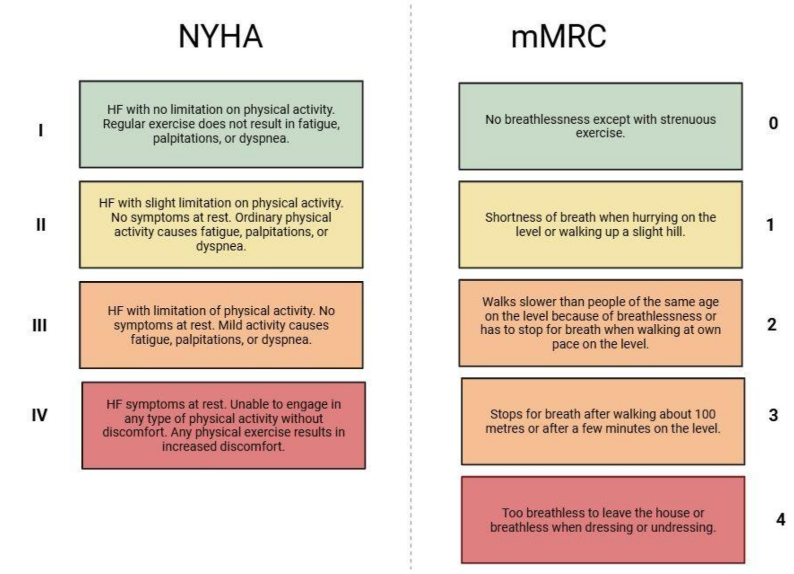

Figure S1. NYHA versus mMRC classification. NYHA, New York Heart Association; HF, heart failure; mMRC, modified Medical Research Council, dyspnea scale. Created with Biorender.com/; accessed on 31st May 2025.

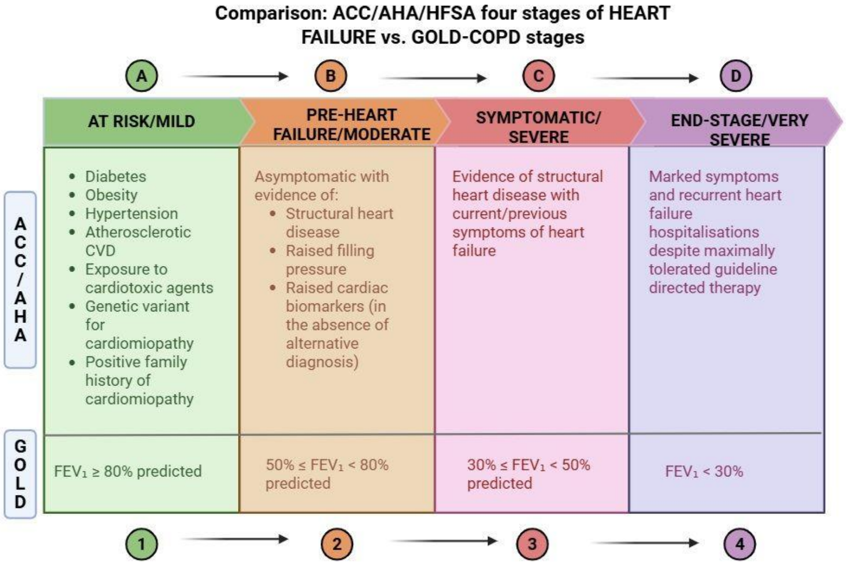

Figure S2. ACC/AHA/HFSA Heart Failure staging, GOLD classification. Comparison. COPD, Chronic Obstructive Pulmonary Disease; FEV1, forced expiratory volume in one second. Created with Biorender.com/; accessed on 9th June 2025.

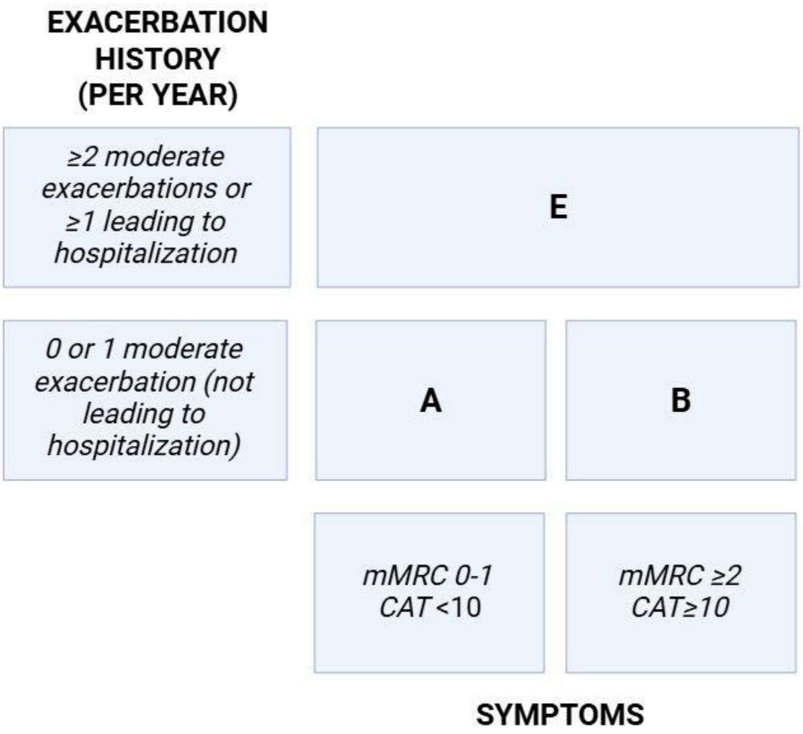

Figure S3. GOLD ABE classification. mMRC, modified Medical Research Council, dyspnea scale; CAT, COPD assessment test. Created with Biorender.com/; accessed on 31st May 2025.

Table S1. Key Diagnostic Tools and Differential Features: HF vs. COPD

Diagnostic Feature / Test	HF	COPD
Onset of symptoms	Often acute or subacute	Gradual, progressive over years
Dyspnea (characteristics)	Orthopnea, exertional dyspnea, paroxysmal nocturnal dyspnea	Expiratory dyspnea, especially on exertion
Cough	Usually absent or mild	Frequent, chronic, productive
Peripheral edema	Common	Rare, unless advanced or cor pulmonale
Pulmonary auscultation	Basal crackles, possible wheezing (cardiac asthma)	Wheezing, rhonchi, diminished breath sounds
NT-proBNP	Markedly elevated (>125 pg/ml outpatient); levels >300–400 pg/ml suggest acute decompensation	Normal or mildly elevated, usually <125 pg/ml; may be elevated in cor pulmonale
Arterial blood gases	Hypoxemia, possible respiratory alkalosis in acute setting	Hypoxemia and hypercapnia common, especially in exacerbations
Spirometry	Possible restrictive pattern during decompensation; usually preserved FEV1/FVC	Persistent post-bronchodilator FEV1/FVC <0.70 confirms diagnosis

Chest X-ray	Cardiomegaly, pulmonary venous congestion, interstitial edema, pleural effusion	Hyperinflated lungs, flattened diaphragm, possible bullae; heart size usually normal
Lung ultrasound	Diffuse bilateral B-lines suggesting interstitial edema	B-lines absent or focal, unless acute infection or pulmonary edema present
Transthoracic echocardiography	Key for diagnosis; may show reduced or preserved EF, increased filling pressures, structural changes	Generally normal; may show RV hypertrophy or pulmonary hypertension in advanced disease
Speckle-tracking echocardiography	Detects subclinical myocardial dysfunction, even in patients with preserved EF; useful for early diagnosis and prognostic stratification in HFpEF	Usually preserved global longitudinal strain; may detect right ventricular strain reduction in cor pulmonale
Cardiac MRI	Provides detailed assessment of myocardial structure, fibrosis (late gadolinium enhancement), edema, and right ventricular function; useful in HF phenotyping and infiltrative cardiomyopathies	Typically normal; may show right ventricular dilation or hypertrophy in advanced disease or chronic hypoxic pulmonary hypertension
Response to diuretics	Rapid clinical improvement and weight loss	Minimal effect
Exacerbations due to infections	Less frequent; triggered by fluid overload or arrhythmias	Frequent; typically triggered by viral or bacterial infections
